# Treatment of High-Polyphenol-Content Waters Using Biotechnological Approaches: The Latest Update

**DOI:** 10.3390/molecules28010314

**Published:** 2022-12-30

**Authors:** Barbara Muñoz-Palazon, Susanna Gorrasi, Aurora Rosa-Masegosa, Marcella Pasqualetti, Martina Braconcini, Massimiliano Fenice

**Affiliations:** 1 Department of Ecological and Biological Sciences (DEB), University of Tuscia, Largo dell’Università snc, 01100 Viterbo, Italy; 2 Institute of Water Research, University of Granada, C/Ramón y Cajal, 4, 18071 Granada, Spain; 3 Faculty of Pharmacy, University of Granada, Campus de Cartuja, s/n, 18071 Granada, Spain; 4 Laboratory of Ecology of Marine Fungi, CoNISMa, Department of Ecological and Biological Sciences (DEB), University of Tuscia, Largo dell’Università snc, 01100 Viterbo, Italy; 5 Laboratory of Applied Marine Microbiology, CoNISMa, Department of Ecological and Biological Sciences (DEB), University of Tuscia, Largo dell’Università snc, 01100 Viterbo, Italy

**Keywords:** polyphenols, biological treatment, bioreactors, water, aerobic granular sludge, conventional active ted sludge, biofilter, photobioreactor, membrane bioreactor, bio-electrochemical systems

## Abstract

Polyphenols and their intermediate metabolites are natural compounds that are spread worldwide. Polyphenols are antioxidant agents beneficial for human health, but exposure to some of these compounds can be harmful to humans and the environment. A number of industries produce and discharge polyphenols in water effluents. These emissions pose serious environmental issues, causing the pollution of surface or groundwater (which are used to provide drinking water) or harming wildlife in the receiving ecosystems. The treatment of high-polyphenol-content waters is mandatory for many industries. Nowadays, biotechnological approaches are gaining relevance for their low footprint, high efficiency, low cost, and versatility in pollutant removal. Biotreatments exploit the diversity of microbial metabolisms in relation to the different characteristics of the polluted water, modifying the design and the operational conditions of the technologies. Microbial metabolic features have been used for full or partial polyphenol degradation since several decades ago. Nowadays, the comprehensive use of biotreatments combined with physical-chemical treatments has enhanced the removal rates to provide safe and high-quality effluents. In this review, the evolution of the biotechnological processes for treating high-polyphenol-content water is described. A particular emphasis is given to providing a general concept, indicating which bioprocess might be adopted considering the water composition and the economic/environmental requirements. The use of effective technologies for environmental phenol removal could help in reducing/avoiding the detrimental effects of these chemicals. In addition, some of them could be employed for the recovery of beneficial ones.

## 1. Introduction

Water is a renewable natural resource, but its inappropriate use has become a problem due to its excessive exploitation caused by the demographic explosion and industrial demands [1]. Environmental pollution is a problem, especially when industrial effluents are discharged into natural water bodies. Most wastes from industries containing organic and inorganic pollutants are easily drawn into wastewater. Many of these pollutants suppose a risk to wildlife species, ecological niches, and ultimately to human health.

**Figure 1:**
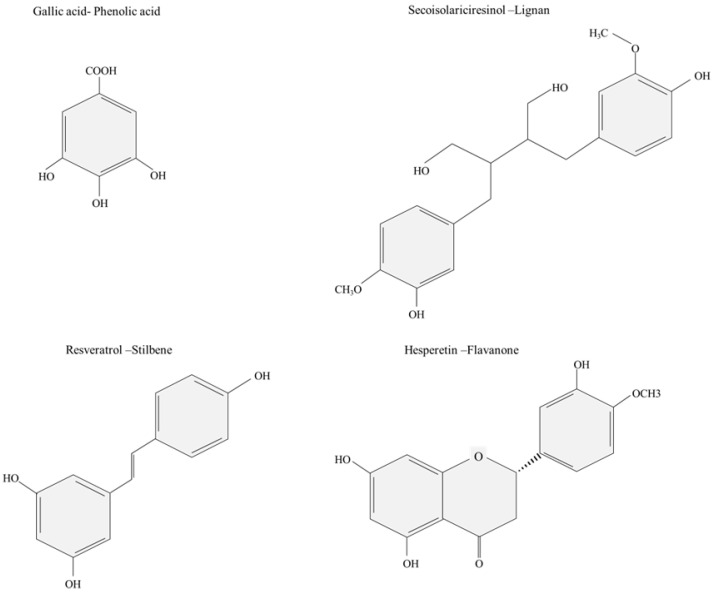
Examples of principal polyphenolic compounds clustered in phenolic acids (gallic acid) [14], lignans (secoisolariciresinol) [3], stilbenes (resveratrol) [15], and hesperetin (flavanone) [3].

Phenol (C_6_H_5_OH) or phenic acid is an organic compound that consists of a phenyl group (-C_6_H_5_) bearing a single hydroxyl group (OH^−^) [1]. Polyphenols are an enormous group of secondary plant metabolites characterized by two or more phenolic rings in their chemical structure [2]. Polyphenols could be mainly classified as flavonoids and nonflavonoids, or according to their complexity, as monomeric and polymeric compounds [3]. However, the principal polyphenolic compounds are grouped into phenolic acids, flavonoids, lignans, and stilbenes [4], characterized by complex chemical structures and the presence of diversified functional groups (Figure 1). For instance, flavonoids are essential bioactive compounds in various biological processes, including nitrogen fixation, photosynthesis, or energy transfer [5,6], while lignans have high pharmacological activities, such as antiviral, antitumoral, and antimicrobial properties [7,8]. Some phenolic compounds have been described as “priority substances” in the field of water policy by the European Water Framework Directive, the Environmental Protection Agency of the United States, and the National Pollutant Release Inventory of Canada [9,10], with 11 out of 126 undesirable chemicals [11]. Phenolic compounds are among the most prevalent water organic contaminants, showing toxicity even at low concentrations and affecting the taste and odor of the drinking water [1]. They are the main substances in the wastewater effluents of several industries, such as pharmaceutical, agricultural, petrochemical, pulp and paper, organic chemicals, and plastic manufacturing [12]. Furthermore, they may lead to water pollution via natural occurrences. In humans, short-term exposure to polyphenol concentrations in the range of 9–25 mg·L^−1^ can cause irritation to the skin and mucous membranes, while long-term exposure may cause lung problems, muscle weakness, tremors, coma, metabolic diseases, and respiratory arrest at lethal doses [10,13]. Actually, it has been described that 50 mg·kg^−1^day^−1^ of bisphenol A is sufficient for human fatality [11].

On the other hand, there is enough evidence that polyphenols and the other bioactive compounds found in widely consumed products (e.g., tea, red wine, cocoa, fruits, fruit juices, and olive oil) have a potentially positive impact on the prevention of a wide range of degenerative and age-related diseases, such as carcinogenesis and tumor development at the cellular level [11,15,16]. For years, polyphenols have been associated with their antioxidant activity, which has been widely verified in vitro. The antioxidant function of polyphenols is provided by their reducing properties, which allow them to act as free radical scavengers [17]. On the other hand, their activity in vivo depends on the bioavailability, the structures of compounds, and the composition and metabolic features of the human gut microbiota. In addition to antioxidant capacity, polyphenols have potential healthy properties for humans as anti-inflammatory, antiallergic, antihypertensive, neuropreventive, analgesic, antiviral, and antimicrobial agents [16,18,19]. These compounds are employed as effective antimicrobial agents in healthcare as an alternative to other treatments and in food preservation [20,21]. For example, the antibiotic activity of phenolic compounds is due to the modification of the bacterial structure, the pathogenic traits, or the changes in metabolism. In particular, tannins and flavonoids are able to inactivate membrane adhesins, enzymes, and transport proteins [22].

Despite the toxic effect of polyphenols on the aquatic environment and wildlife, the benefits are principally related to their consumption which allows for the prevention of diseases in humans and food preservation. This makes it essential to know the right approach for selecting and extracting the polyphenol of interest from the various effluents. These waters will be later treated by biological technologies to remove the remaining pollutants, giving the place a sustainable and circular economy, recovering the added-value compounds, and discharging high-quality waters into natural water bodies.

## 2. Polluted Waters as Sources of Phenolic Compounds: The Challenge of Their Profitability in the Circular Economy

The presence of polyphenols in water could be due to natural or anthropogenic sources. The decomposition of vegetal material (fruits, vegetables, or resins) contributes to the phenolic concentration in natural waters due to their water solubility. In drinking waters, the pollution is mainly due to the lixiviation of residues, while in lakes and other water bodies, this occurs by in situ decay. However, the water quality is primely injured by the pressure exerted by the discharge of industrial wastewater. Polyphenols are present in the effluents from several industrial activities, such as pharmaceuticals, plastics, paint, pulp and paper, agrofood manufacturing, petrochemical, and wood products [3]. In fact, their concentration reaches 3900 mg·L^−1^ in coking operations, 6800 mg·L^−1^ in coal processing, 1220 mg·L^−1^ in the petrochemical industry, 500 mg·L^−1^ in refineries, or 1000 mg·L^−1^ in the agrofood industry (e.g., in olive washing waters) [23,24,25]. Table 1 shows some effluents containing polyphenols from some industries.

The presence of polyphenols in industrial wastewater effluents represents a major environmental problem related to the toxic effects on microorganisms, plants, and animals in the trophic chain [29]. However, the management of these harmful compounds could become a challenge for their valorization within a circular economy. Interesting sources of polyphenols are the wastes and byproducts from the agrifood production processes that could be employed in the food preservation, cosmetic, and pharmaceutical industries, supposing a new sustainable way to obtain the functional compounds could be found [17,30]. Therefore, the recovery of these substances from wastewater meets the two-fold objective: biodegradability improvement and byproduct valorization [31].

For example, the production of olive oil and wine is one of the most important economic activities in Mediterranean countries. These activities generate large amounts of solid and liquid wastes containing high levels of polyphenols [24,32]. Therefore, their recovery from these wastes provides an opportunity to obtain valuable biomolecules with a concomitant reduction in environmental toxicity issues [29,32]. In olive mill wastewater (OMW), polyphenols are partitioned between the water and oil phases, but the major fraction is retained in the wastewater due to the high solubility in water. Polyphenol extraction from OMW is very promising since these compounds are potent antioxidants. Among them, hydroxytyrosol and tyrosol are postulated as the most economically relevant phenolic components in the cosmetics, food preservation, and pharmaceutical industries [29,33].

## 3. Recovery of Polyphenols from Wastewater

Phenol recovery is necessary and useful but difficult, especially when unknown interactions with other pollutants in wastewater may occur [34]. However, this procedure offers a great economic advantage and makes these wastewaters less hazardous and easier to treat using biological methods [33]. Therefore, extensive research efforts have been directed toward the recovery of polyphenols from some agrofood industries (such as OMW or wineries) wastewaters [31,33].

The recovery of polyphenols is mostly performed by membrane processes, liquid–liquid extraction, which is often assisted by surfactants, or solid phase extraction using diverse resins for their recovery [29]. For example, the use of membrane processes requires small areas, and the separation of specific compounds could also facilitate the subsequent biological wastewater treatment [31,35]. The diverse membrane characteristics (ultrafiltration, nanofiltration, and reverse osmosis) could allow for the retention of solutes on the membrane surface, resulting in material accumulation (biofouling) that interferes with the concentration polarization, reducing the membrane lifetime [31]. Another example is the solid-phase extraction procedure for the recovery of phenolic compounds using several resins. The results of these studies highlighted how the desorption process is difficult because the solvents have low selectivity for specific polyphenols, and polyphenol ionization limits the removal performance [34].

Nowadays, much effort is put into the search for more efficient alternatives to extract the most interesting polyphenols for the cosmetic, pharmaceutical, and food industries. Currently, the adsorption process on suitable materials is postulated as an efficient method to recover specific polyphenols from aqueous solutions [36]. A large selection of synthetic polymeric adsorbents (such as polystyrene-divinylbenzene or divinylbenzene-ethylvinylbenzene copolymers) is available for carrying out an optimal process [36]. The adsorption on resins is a common separation technology, as the relatively inexpensive resins are lasting and chemically stable. In addition, these technologies include liquid extraction with a deep eutectic solvent, deionized water, or aqueous solutions of cyclodextrins using a semiautomatic extractor and multielement mixed micelle. The adsorption system is considered an economical, simple, and reversible method which avoids the use of toxic solvents. One of the most interesting and well-known materials used for adsorption is activated carbon [37,38]. The technique with activated carbon has been tested with several sorbents, including methylene blue and manganese oxide [39,40]. However, the latest research employed animal proteins as coating biopolymers, avoiding environmental problems [33].

## 4. Technologies to Remove Phenolic Compounds from Water Sources

Considering the harmful effects of some polyphenols and their derivatives on human health and natural environments, the removal of these compounds from water and wastewater is a great challenge for human and environmental safety, and the search and recognition of effective tools for their treatment, degradation, and removal are worthwhile [2,11]. Accordingly, great efforts have been spent, using physical, chemical, and biological methods to develop and optimize techniques for finding a sustainable solution to this problem. Undoubtedly, physicochemical technologies are more expensive than biological ones, and sometimes they are not completely effective because undesirable compounds are often produced as intermediates [41].

Among the physical-chemical methods, conventional processes have been applied for phenolic compound removal, such as distillation, adsorption, solid-phase extraction, liquid–liquid extraction, and catalytic and wet air oxidation. Furthermore, the last decade has been characterized by the development of phenolic acid degradation technologies. Some of these technologies are based on advanced oxidation, membrane filtration, heterogeneous photocatalysis, ozonization, enzymatic treatment, and the Fenton reaction [1,4]. However, these technologies have serious disadvantages, which are mainly related to their economical cost (e.g., the high energy consumption) and environmental unsustainability due to the production of secondary pollutants [42]. For these reasons, the search for biological alternatives for the treatment of effluents polluted with phenols is a challenge of great biotechnological interest.

The biological technologies for degrading and removing phenolic compounds are considered to represent a promising alternative because their main advantage is phenol degradation into nontoxic products, or even mineralization, accompanied by lower exploitation expenses [23]. Hence, there is a vast demand to develop economical, green, and sustainable technologies, in particular for rural areas with low income and highly impacted by agrofood industries [24] and/or in petrochemical, paper, and pharmaceutical factories [10,43].

It is very attention-grabbing to observe that the main development of biological technologies to remove phenolic compounds was based on aerobic conditions [43,44]. However, in recent years more attention has been paid to the development of anaerobic systems due to their tolerance to high organic loading rates (ORL), lower sludge excess production, and the valorization of methane generation [45,46]. Moreover, one of the most relevant advantages of anaerobic processes is robustness and resilience against changes in the influent composition [47].

The biological treatment of wastewater with high polyphenol content could be seriously compromised, especially when industrial discharges contain flavan-3-ols, flavonols, and tannins due to their wide spectrum of antimicrobial activities [48]. Although biological treatments are described as affordable, efficient, and environmentally friendly processes, the stability of the process could be compromised by the high load of phenolic compounds and their inherent toxicity [24]. Thus, it is crucial to develop technologies adapted to water composition and origin. In addition, it is important to allow for the adaptation of the initial biomass (inoculum) because microbiota with high activity and robustness are essential for achieving the efficient removal of recalcitrant compounds, mitigating the effect of wastewater discharges into natural environments [49].

## 5. Feasible Biological Technologies for the Removal of Phenolic Compounds from Waters

**Figure 2:**
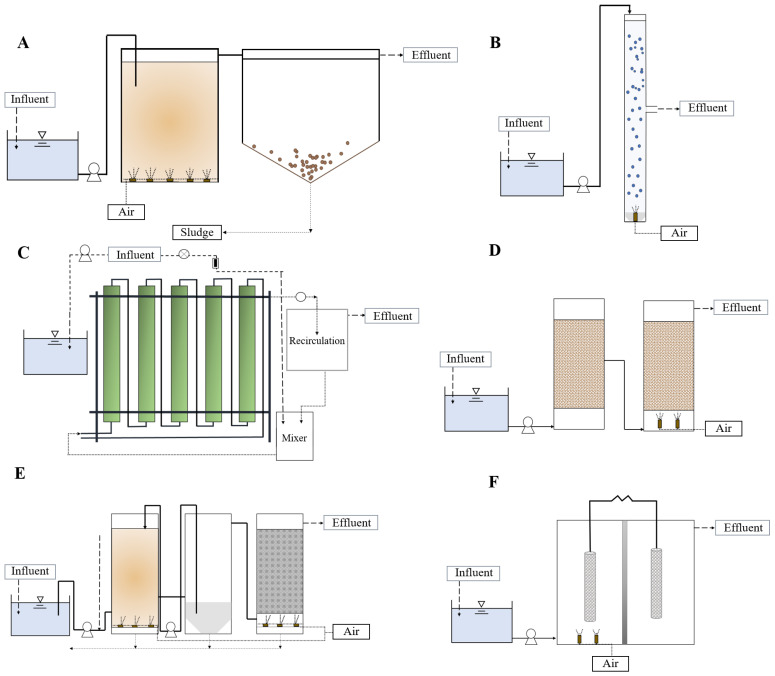
Diagram of biological water technologies for removing phenolic compounds from water: conventional activated sludge (**A**); aerobic granular sludge (**B**); photobioreactor (**C**); biofilter (**D**); membrane bioreactor (**E**); microbial fuel cell (**F**).

The optimization of biological processes poses attractive alternatives to be implemented, because they are usually eco-friendly and economical, minimizing the generation of subproducts while being generally easy to operate. The microorganisms used in biological technologies may promote the degradation of several organic and inorganic contaminants found in wastewater. The biodegradation could be mediated by extracellular or intracellular enzymes, and the pollutants could be removed by extracellular biosorption or active uptake and then incorporated and bioaccumulated [50,51,52]. Biological treatment could encounter difficulties in removing recalcitrant products, but the removal of polyphenols from water using many technologies has been reported [53]. The most useful technologies for the degradation of phenolic compounds based on biological treatments are discussed hereafter (Figure 2).

### 5.1. Conventional Activated Sludge

Conventional activated sludge (CAS) is a biological technology that has been used for treating wastewater for more than 100 years [54]. In the ‘classical’ context, CAS is a process performed by a suspended microbial biomass under aerobic conditions; the cells in contact with polluted water oxidize the compounds within an aerated chamber, and the biomass-liquid phases are then separated into a second chamber that acts as a settler. The biomass of the activated sludge is composed of large flocs with filamentous microorganisms [55].

The activated sludge-based technology is the most diffused wastewater treatment worldwide [56]; thus, it is essential to understand which effects (on the global process) are exerted by the presence of phenolic compounds on raw water (inlet) in order to comprise the feasibility of their application to high-polyphenol-content waters. Siripattanakul-Ratpukdi et al. [57] investigated CAS system inhibition due to the high presence of phenolic compounds using synthetic wastewater supplemented with phenol as the sole carbon source to simulate the contaminated wastewater. The results pointed out that the system was able to completely remove the phenol in the case of low–moderate contamination (10 mg·L^−1^ phenol), whereas a low phenol removal ratio was observed in the highly contaminated scenario (100 mg·L^−1^) due to the inhibition caused by its recalcitrant nature. Moreover, the authors highlighted that phenol adsorption might also occur due to physical-chemical properties, such as the porous floc structure, which increases the surface area in contact with the wastewater and facilitates the adsorption process [58].

More recent studies reported the effective treatment of olive oil mill wastewater (OOMW) with a total phenol concentration of ~8730 mg·L^−1^ using CAS, but only after a dilution of up to 128 mg·L^−1^ of polyphenols, obtaining a chemical oxygen demand (COD) and polyphenols removal ratio of 90% and 92%, respectively [59]. However, it is worth noting that various local and or international regulations do not permit effluent dilution [60,61].

However, CAS showed several disadvantages in comparison to novel technologies, such as the production of excess sludge, the big surface needed, or the low biomass retention time [62,63].

In recent years, the CAS configuration has been improved to overcome the technology’s weaknesses. For instance, integrated fixed-film activated sludge (IFAS) is one of the most promising recent CAS configurations for its advantages, such as longer solids retention time, nutrient removal, complete nitrification, and lower carbon footprint [64]. Ahmed et al. [65] designed a kinetic model to evaluate the IFAS efficiency in petrochemical wastewater treatment and demonstrated that this technology is a viable bioprocess but is sensitive in non–steady states. Similarly, Lin et al. [66] carried out a phenol biodegradation experiment using a biofilm reactor, obtaining removal rates of 94–96% at different hydraulic retention times (HRT); moreover, they corroborated the results with their own mathematical model.

### 5.2. Aerobic Granular Sludge

Aerobic granular sludge (AGS) is a recent biological technology characterized by the immobilization of cells embedded in a tridimensional polymeric matrix with a spherical shape. AGS is usually operated in aerated cylindrical columns to promote the compactness and density of the granules, allowing for high biomass retention and resistance against toxic compounds. In fact, toxic compounds do not affect the single cells because a gradient is generated from the external to the internal layers, permitting high resilience against recalcitrant pollutants [63]. Several researchers have focused on the treatment of phenol [67], *p*-nitrophenol [68], halogenated phenols [69,70], and phenolic acids [24]. The microbial consortia used in the process were able to degrade the toxic compounds by diverse metabolic pathways, although it has been demonstrated that a preliminary adaptation of the granular sludge in the presence of phenols improves the removal performance [68].

The successful application of AGS for degrading phenolic compounds has been extensively demonstrated during the last decade [24]. Firstly, Liu et al. [71] and Jiang et al. [72] cultivated granular biomasses in presence of phenols to establish their degradation rate and toxicity at different levels/concentrations. Liu et al. [71] concluded that, despite the damage that polyphenols could cause to the granular biomass, the polyphenol removal activity of granular sludge was positively correlated with higher biomass concentration (Mix Liquor Suspended Solids, MLSS). For the first time, Jiang et al. [72] demonstrated the capability of aerobic granules to provide stable and efficient phenol biodegradation, remarking that the granules represent an excellent immobilization strategy and biofilm functional structure, overcoming the inhibition effect of phenols on microbial growth and biodegradation activity. Undoubtedly, this research reported that the phenol concentration affects the selection of the phenol-degrading microorganisms within the granular biomass and directly impacts the robustness of the system and, consequently, the ability to achieve high performance.

Later research described several operational conditions and designs to optimize the removal of phenolic compounds. For instance, one research revealed that *p*-nitrophenol could be successfully removed; even an increase in *p*-nitrophenol concentration led to an increase in the performance rate (operating with values up to 40.1 mg·L^−1^) [68]. Concomitantly, the start-up and optimization of AGS technology for operation in continuous flows posed a challenge for overcoming the possible disadvantages that batch-flow operations can entail at the industrial level. Jemaat et al. [44] investigated the response of AGS in a continuous-flow treatment of industrial wastewater in sequentially alternating pollutant scenarios using *p*-nitrophenol, phenol, and 2-chlorophenol with a concentration of 15 mg·L^−1^. Although these loads were lower than those used by Suja et al. (25–200 mg·L^−1^) [68] and Wang et al. (10–50 mg·L^−1^) [69], the difficulties involved in the continuous flow and the need to degrade o-cresol generated greater complications for the steady-state of granular biomasses. The results showed that AGS, in a continuous flow, was able to remove phenol and *p*-nitrophenol. During the first months of operation, the HPLC analysis did not detect *p*-nitrophenol in the effluent, but *p*-nitrocatechol (an intermediate of the aerobic degradation pathway) was found. Nonetheless, after this period, *p*-nitrophenol and *p*-nitrocatechol were below the detection limit of HPLC, and the nitrogen and o-cresol removals were almost complete. During the last stage, the system showed notable flaws, such as the serious damage to biomass conformation, a reduction in organic matter removal, the detection of intermediate compounds, and the accumulation of 2-chlorophenol. These issues suggested a need for further research to solve the inefficiency of AGS in continuous flow [44].

In addition, some authors tested a strategy based on the use of easily degradable carbon sources as co–substrates to hardly biodegradable polyphenols to ensure the stable biodegradation of these xenobiotics. Wang et al. used glucose as a co–substrate of 2,4-dichlorophenol to promote granule formation, obtaining successful polyphenol removal and biomass stability for long-term operations [69]. Ho et al. [67] reported that, in the presence of co–substrates as an additional carbon source, phenols at concentrations up to 3000 mg·L^−1^ were removed with minimal lag time, while at 5000 mg·L^−1^ phenol concentration, the granular biomass exhibits a longer lag time (20 h). In addition, this research demonstrated that granular sludge systems had weaker inhibition than CAS systems for treating phenol-rich wastewater.

The improvement of AGS technology for treating phenolic compounds has greatly increased during the last decade, driven by a greater knowledge of how to promote the metabolic pathways of the microorganisms of interest. Muñoz-Palazon et al. [24] operated AGS in sequential batch reactors with a mix of phenolic acids at concentrations from 50 to 300 mg·L^−1^, achieving excellent removal performance. Furthermore, the authors noted that higher concentrations (600–1000 mg·L^−1^) meant a strong detriment for the physical-chemical and polyphenolic removal rate (ranging from 30–60%). In this sense, despite the presence of the co–substrate as an alternative carbon source, it was observed that the proliferation of filamentous microorganisms promoted the breakage and disintegration of granules, resulting in floc-biomass formation and the destabilization of the steady state.

### 5.3. Photobioreactors

Photobioreactor technology represents a potentially viable strategy for removing pollutants from sewage, producing, at the same time, microalgal biomasses that are considered valuable products [73]. This process has demonstrated high photosynthetic efficiency and a low footprint, offering an environmentally sustainable alternative versus physical-chemical technologies that produce chemical waste or sludge as byproducts [73,74]. This technology is based on the growth of microalgae in illuminated reactors, which could effectively transform the inorganic nutrients into organic compounds by means of photosynthesis [73]. Microalgae can be co–cultivated with bacteria to optimize phenolic compound removal; microalgae produce the oxygen required by bacteria to mineralize the organic pollutants, and the carbon dioxide produced by bacteria is, in turn, used by the microalgae [75]. Therefore, the photosynthetically produced oxygen avoids the insufflation (in the reactor) of the air needed to support the bacterial metabolic processes [76]. Biofilm photobioreactors involve microalgae–bacteria consortia and show several advantages, such as high biomass concentration, high biodegradation ratios, and resistance against toxic compounds [76]. Likewise, the biofilm promotes mass transfer from the external to the internal layer, providing hypoxic niches for the growth of facultative or strict anaerobic microorganisms [77]. The photobioreactor design needs to be adapted to the composition of wastewater and the operational conditions. For instance, Muñoz et al. [75] tested different photobioreactor configurations in order to achieve maximum efficiency in polyphenol removal. The reactors were based on four designs: a flat plate and a tubular packed-bed photobioreactor with the algal-bacterial biofilm attached to a glass material; a flat plate and tubular photobioreactor with the biofilm attached to the bioreactor walls; an algal-turf reactor open pond with biofilm attached on the reactor base, and a column photobioreactor with suspended biomass. All of them operated under a continuous illumination irradiance at 180 µE·m^−2^s^−1^. Muñoz et al. [75] indicated that photobioreactors involving biofilms presented two limitations that caused operational problems: photoinhibition, by the high density of the photon flux density, and the potential risk of clogging due to the excess biomass. In addition, the bioreactors with a suspended biomass and biofilms achieved similar removal efficiencies, but the latter promoted better biomass settleability than the suspended cultures.

Maza-Márquez et al. [77] described the treatment of real olive-washing wastewater (OWW) using a photobioreactor designed as a semi–open system consisting of a mixing tank, a tubular photobioreactor (composed of five-column tubes), a recirculation tank, and a collector tank. In this study, the average light intensity was 450 ± 50 µE·m^−2^ at the outer layer, but the dark color of the OWW reduced the light transmission and may have inhibited the growth of the microalgae. Even so, the concentration of the polyphenols was drastically reduced (90.6%), revealing that the algae–bacteria consortium improves the metabolisms responsible for phenolic compound degradation, which was also corroborated by Chan et al. [76]. Moreover, the biological oxygen demand (BOD_5_) was completely removed in all scenarios. Therefore, this research supported the fact that microalgae provide enough oxygen for the bacterial degradation of polyphenols.

The previously described photobioreactor was implemented at full-scale [78], highlighting the success of the technology for removing COD and BOD_5_ from OWW_._ Further, the results of spectrophotometric analysis by Folin–Ciocalteu showed the almost complete removal of the phenolic compounds, which was related to the microalgae–bacteria consortia composed of *Rhodopseudomonas* and *Azotobacter* bacteria and *Sphaeropleales* microalgae [78].

### 5.4. Biofilters

Biofilter technology is one of the most important biological processes that is used for the removal of organic pollutants. In fact, biofilter technologies are widely employed for providing drinking water [79]. Biofilters are mostly characterized by the ability to separate the particulate matter and biomass from the aqueous phase, reducing the natural organic matter and several compounds. Biofilter performance allows for a reduction in the taste and odor of water, as well as removing the micropollutants involved in environmental concerns and human health [80]. Any type of support material that allows biomass immobilization could be used in biofilters. Nowadays, several materials of both natural (rock, slugs, sand, anthracite, and granular activated carbon) and artificial (membranes and plastics) origin are used as carriers [81,82,83]. However, the ‘classical’ biofilter technology is based on the use of filter sand of different particle diameters from a rapid-rate to a slow-rate [84]. In addition, the biofilter process is very attractive because it significantly reduces the chlorine demand [79]. Usually, the proliferation of microbiota within an immobilized biofilm depends on the influent composition, inoculum origin, material support, and operational conditions. The versatility of this technology makes it difficult to define a universal reactor design since an effective design requires deep initial knowledge and the characterization of all the parameters to be considered (inlet water characterization and the required quality). Current molecular biological techniques have allowed for the promotion of microorganisms of interest to degrade target pollutants [79].

Although biofilter technology is not so widely used for the treatment of high-polyphenol-content waters, Huang et al. [49] have tested the treatment of wastewater with high content of a complex mixture of polyphenols (2,5-diethylphenol, trimethylphenol, ethylphenol, 2-(1-methylpropyl)-phenol, 2-ethyl-6-methylphenol, and 1-ethyl-4-methoxyphenol). The composition of the influent water was even more complex due to the presence of other kinds of biorefractory compounds widely found in coking wastewater, which implied more difficulties for polyphenol removal by biological treatment. The results revealed that after anaerobic filtration treatment, the phenols were quite degraded, and the achieved removal rates were 92.31% for *p*-diphenol and 53% for 2,5-diethylphenol. Additionally, the chromatography analyses showed that new compounds (such as dihydroxybenzene and cyclohexanone) were produced during the bioprocess, indicating the presence of intermediate molecules or simple compounds obtained from the full or partial degradation of the polyphenols. The different heights along the biofilter generated a gradient of pollutants, subproducts, and oxygen conditions that affected the microbial community structure responsible for the biotransformation and biodegradation processes [49,82].

### 5.5. Various Treatments Combined with Biofilters

#### 5.5.1. Conventional Activated Sludge Coupled with an Immobilized Biological Filter

As mentioned before, CAS technology is the most implemented biological technology for treating urban wastewater [85]. CAS includes an aeration chamber and a secondary clarifier; in the first chamber, the raw wastewater is mixed with the floc microorganisms, where the aerobic niche promotes the fast degradation of organic matter. Then, the water is transported to the secondary clarifier, where the floc-forming bacteria and the particulate matter are settled in order to provide a clear effluent. However, CAS efficiency for the removal of suspended particulate matter in the secondary settler is quite limited; thus, the coupling of CAS with a subsequent biofilter could improve the retention of the suspended solids and the solid-water phase separation. Additionally, the water streamflow through the reactor allows for the removal of other substances due to the development of different niches along the biofilm that is colonized by microbial communities with different metabolic competencies. The secretion of sticky and negatively charged extracellular polymeric substances by the bacterial biofilm makes it more resistant to toxic compounds; in addition, the biofilm promotes the mass transport and substrate conversion from the external to internal layers [86,87]. Thus, the toxic effect of polyphenolic compounds could be weakened along the depth/height of the immobilized biofilm, and the single cells are not directly affected, as they are embedded in a polysaccharide membrane that protects them. The implementation of conventional biological processes coupled with biofilter technology has been deeply studied for treating industrial wastewater with high-volatile organic compound content, harmful gases, and numerous toxic compounds to avoid the severe contamination produced by these industries [88]. Some microorganisms that form the attached biomass were able to convert target pollutants into intermediate or final products with low or absent harmfulness.

Tong et al. [89] described a pilot-scale CAS process coupled with an immobilized aerated biofilter designed for treating wastewater characterized by a large number of recalcitrant compounds that are generated during the oil extraction process. The carrier employed for the biofiltration process was the polycin urepan, which was designed with micro- and macropores to immobilize a wide spectrum of microorganisms of several sizes. The treatment train consisted of a CAS system followed by a settling tank; then, the water streamflow was passed through an immobilized biological aerated filter (I-BAF). The composition of the oil wastewater was very complex due to the low organic substrate concentration, the high presence of refractory organic compounds, and a low C:N:P ratio (which was 100:6:0.007, whereas it should be close to 100:5:1 to achieve good performance). Phenolic compounds were the most prominent group (with 15 kinds/species), accounting for ca. 31.5% of the load influent. The results revealed that CAS technology was able to remove all phenolic compounds without the need to pass through an I-BAF. The bacteria consortia identified in the activated sludge were more diverse than those on the biofilm of the I-BAF. Tong et al. [89] highlighted the role of *Pseudomonas* sp., *Planococcus* sp., and *Bacillus* spp. in phenolic compound removal; their role in polyphenol degradation in various natural and engineering environments has been studied in-depth [90,91,92].

#### 5.5.2. Expanded Granular Sludge Bed Coupled with Biofilter

Expanded granular sludge bed (EGSB) technology is considered to represent the third generation of anaerobic reactor technologies. This process is characterized by the expansion of granular sludge and the promotion of the mass transfer between pollutants and microbiota by the high recirculation rate and a high height/diameter ratio (≥20) [93]. Some authors combined the EGSB with biofilters for the treatment of recalcitrant compounds because the fixed biofilm promotes syntrophic mechanisms and resists and degrades polyphenols and their intermediates of degradation [93,94]. Several strategies for an EGSB coupled with biofilters have been tested. For instance, Wang et al. [93] implemented an aerobic biofiltration system to minimize the surface needed for the implementation of the treatment, but the exploitation cost increased due to the requirement of air insufflation. Rintala and Puhakka [95] reported the effectiveness of the EGSB coupled with an aerobic biofilter for chlorophenols degradation, demonstrating that the molecules are anaerobically attacked and dechlorinated, allowing for their mineralization in the subsequent aerobic biotreatment step. However, Collins et al. [94] highlighted the economical disadvantages related to this system (operations carried out under aerobic and mesophilic conditions). Therefore, these authors operated the EGSB combined with an anaerobic biofilter (ABF) at lower temperatures (15 °C). This system had a long start-up period, but once the steady–state was achieved, no trichlorophenols or 2,4 dichlorophenols were found in the effluent, evidencing the successful dehalogenation and a split reductive dichlorination pathway. In the subsequent scenarios, Collins et al. [94] added a higher polyphenol concentration to the influent, and they observed a similar trend. The HPLC results revealed the absence of the phenols and the presence of unidentified compounds possibly correlated with 4-hydroxybenzoic acid or other intermediate degradation products [96].

#### 5.5.3. Membrane Bioreactor Technology

Membrane bioreactor (MBR) technology combines the activated sludge process with a membrane to separate the solid-liquid phases, avoiding the need for a secondary settler [97]. The configuration of the MBR has been developed and optimized in order to increase performance and removal efficiency. Depending on the required quality of the effluent, the employed membrane can be of nanofiltration, microfiltration, or ultrafiltration [98]. In the ‘classical’ MBR design, the configuration can generally have a submerged or side-stream membrane using external recirculation. Currently, novel changes have been implemented in the classical MBR designs to adhere to more restrictive legislation [99,100,101]. There are several ways to remove the phenolic compounds contained in wastewater by diverse membrane bioreactor configurations [11], including the moving-bed bioreactor (MBBR) [100,102], the anaerobic–anoxic–oxic (A^2^O) membrane reactor [25], the two-phase partitioning membrane bioreactor [101], or the few novel designs with photocatalysis, using material coupled to the MBBR [103].

During the last two decades, the interest aroused by membrane technologies led to the development of many novel configurations optimized for obtaining maximum polyphenol removal yields. Hosseini et al. [102] used the MBBR for treating phenolic compounds at concentrations in the range 200–800 mg·L^−1^, with HRT from 8 to 24 h. The results suggested that, regardless of the HRT, the ratio of phenolic COD:total COD should be close to 0.6 to achieve successful removal. The authors emphasized that MBBR technology was able to remove 480 mg·L^−1^ phenol COD in short cycles (8–12 h), and the biomass exhibited low sensitivity against toxic load [102]. On the contrary, the A^2^O MBR system used to treat coal gasification wastewater had strong and negative effects caused by changes in HRT. The water inlet had high phenolic (1000 to 1600 mg·L^−1^) and COD (2000 to 4200 mg·L^−1^) concentrations. The real origin of the wastewater made the composition highly variable, with poor biodegradability due to the presence of refractory compounds [25]. Even so, the technology was able to remove almost all COD and ammonium (97% and 92%, respectively), as well as the total phenolic compounds (from the initial concentration of 776 to 2.32 mg·L^−1^). The authors described that the refractory compounds were converted into biodegradable substances in the anaerobic chamber and, subsequently, microbial activity notably reduced the compound toxicity in the anoxic and oxic chambers. In this case, if it was mandatory to meet a requirement for nitrogen discharges into water bodies; A^2^O MBR could be an optimal option for such implementations.

More recent advances have been achieved by combining the membrane bioreactors with other technologies. Mancuso et al. [103] coupled a TiO_2_-based-photocatalyst that was doped with Fe and/or Cr with an MBBR. They emphasized the difficulties arising from OMWW treatment using only MBR technology due to the short membrane lifetimes, biofouling clogging, and high operational costs [99,104]. Therefore, they proposed a preliminary photocatalysis treatment driven by solar UV in presence of a TiO_2_ semiconductor and metal elements to save energy and solve the limitations given by the energy required for the operation. The results highlighted high polyphenol removal efficiency (close to 97%), as well as a notable increase in the biodegradability of these compounds in the presence of H_2_O_2_ and the consequent improvement in the capability of treating larger wastewater flows.

Most of the industries related to chemical and agrifood factories produce effluents with high levels of salinity (representing 5% of the total industrial wastewater production), which directly affects biological processes due to osmotic pressure damaging the cells. Few studies have investigated the impact of salinity fluctuations on various aerobic processes in membrane technologies [105]. Muñoz Sierra et al. [46,106] particularized the lack of knowledge about the anaerobic MBR treatment of wastewaters with high polyphenols content and variable salinity. The research demonstrated an efficiency of 86–98% in terms of total phenolic compound removal in the presence of 18–37 gNa^+.^L^−1^. At these salinity concentrations, the biomass showed robustness and resilience against adverse conditions, but at higher concentrations, methanogenic activity and cell membrane integrity were compromised. Besides, an extractive membrane bioreactor (EMB) was implemented to treat landfill leachate effluent with a high phenolic concentration (150–200 mg·L^−1^ [107]. The technology was able to reduce the phenolic fraction by ~98%; in addition, the HPLC analysis corroborated the absence of polyphenols and intermediate metabolites [107].

### 5.6. Microbial Bio-electrochemical Technology

Microbial bio-electrochemical technology (MET) is a growing biotechnological tool that combines electrochemistry, material science, and microbiology with the aim of generating energy [108].

The most used configuration of MET is the microbial fuel cell (MFC) technology based on converting the chemical energy of microbial metabolism into electricity during water treatment [109]. The process consists of an anode chamber and a cathode chamber separated by a proton exchange membrane or a salt bridge [110]. In the anode, microorganisms oxidize the substrates and produce electrons and protons. The protons are conducted to the cathode through the exchange membrane, and the electrons are transported to the external circuit [110]. The membrane prevents the transfer of bacteria from the anode to the cathode and limits oxygen diffusion in the opposite direction [111]. Although the MFC is the most well-known design in METs, currently, the optimization and development of METs, combining catalytic redox activity with different abiotic electrochemical parameters and physic conditions, is in continuous expansion [112,113]. The versatility of the designs and the operational conditions, the consumption of several biodegradable substrates, and the production of energy make this technology one of the greenest for water treatment [114].

Friman et al. [115] implemented a bio-electrochemical cell technology to degrade phenols, operating similarly to MFCs, but with a constant external voltage applied between the electrodes. The phenol removal rate was ~80%, and the authors observed that it was not proportional to the time of contact between the pollutants and biomass, but higher biomass concentration was supposed to increase phenol removal efficiency. Hedbavna et al. [116] described how phenolic compounds found in the groundwater (caused by refinery industry lixiviate) could serve as electron donors for voltage production, while the microorganisms found in the anode could be electron acceptors. The MFC systems were tested using phenolic compounds and acetate as electron donors, with closed and open circuits, and the results were similar for both the MFCs, with a polyphenolic reduction > 86% (from 1440 to >200 µM). During the initial phase, 4-hydroxy-3-methylbenzoic acid and 4-hydroxybezoic acid (two metabolites of phenols) were detected at high concentrations in the anode chamber (108 µM and 104 µM, respectively). However, after stabilization, the values began to gradually decrease, indicating an increment in intermediate metabolite degradation.

The phenols were able to relatively inhibit electricity production (35–65%), but the robustness of the technology demonstrated high degradation rates for phenolic compounds such as 4- hydroxibezoic, syring, and vanillic acids, and their intermediate metabolites (despite the inherent toxicity of polyphenols) [117]. Li et al. [117] reported that the absence of additional electron donors improved the removal efficiency of phenolic compounds, as the microbiota was driven to use them as carbon sources. The research demonstrated that the removal of the phenolic compounds was driven by the degradation process rather than the adsorption process and that the microorganisms used intermediate metabolites for energy production and voltage generation instead of the parent phenolic compounds [117]. Li et al. [117] established that the fermentative process was not inhibited by high concentrations of phenolic acids; in addition, the main biotransformation of syringic acids gave rise to 3,4-dihydroxy-5-methoxybenzoic acid, which was subsequently converted into gallic acid and pyrogallic acid [118].

## 6. Microorganisms and Their Products Involved in Phenolic Biodegradation

Microorganisms are responsible for the suitable performance of all biological technologies described previously. A high number of microorganisms are able to degrade polyphenols in aquatic environments, including bacteria, fungi, and protozoa. Efficient biodegradation could be carried out by a single species [119,120] or microbial consortia [24,77]. During the design of a bioprocess, the selection of microorganisms or microbial consortia according to the nature of the water and the target pollutants is essential. The ability to achieve high performance will depend on the contaminant nature and the metabolic possibilities of the biomass in the bioreactor [91]. However, some drawbacks are found in urban or industrial wastewater, given the complexity of the matrices. Therefore, the microbial consortia employed as inoculum are generally selected by the technology configuration and influent composition since some microorganisms have competitive advantages in consuming, digesting, or degrading compounds under different abiotic and biotic parameters.

Given that phenol is widespread, some microorganisms have developed metabolic pathways for using it as carbon and/or energy source in several environments [1]. Undoubtedly, some microbial groups are more resistant to phenolic compounds. The microorganisms that are able to degrade polyphenols have been recognized in both the *Bacteria* and *Eukarya* domains. Among the best-known microorganisms that can successfully carry out phenol biodegradation, there are bacteria such as *Bacillus*, *Pseudomonas*, *Flavobacterium,* and *Alcaligenes*, as well as fungal representatives, such as *Trichosporon*, *Aspergillus*, *Trametes,* and the yeast *Candida tropicalis* [24,121].

**Figure 3:**
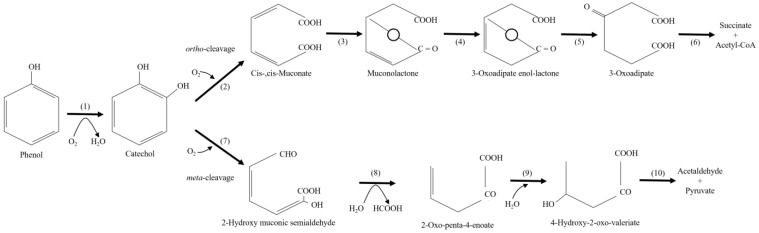
Aerobic phenol biodegradation pathways. (1) phenol monooxygenase, (2) catechol 1,2-dioxygenase, (3) muconate lactonizing enzyme, (4) muconolactone isomerase, (5) oxoadipate enol-lactone hydrolase, (6) oxoadipate succinyl-CoA transferase, (7) catechol 2,3-dioxygenase, (8) hydroxymuconic semialdehyde hydrolase, (9) 2-oxopent-4-enoic acid hydrolase, (10) 4-hydroxy-2-oxovalerate aldolase [125].

Aerobic bacteria transform phenol into nontoxic intermediate compounds that enter into the tricarboxylic acid cycle through ortho- or meta-pathways of degradation (Figure 3) [12]. In both pathways, phenol is firstly converted into catechol by the monohydroxylation of the C_6_ ring at the *ο*-position, which is catalyzed by phenol hydroxylase [91]. Phenol hydroxylase catalyzes the attachment of a hydroxyl group at the ortho-position of the aromatic ring, and then phenol is converted into catechol. This reaction is carried out by NADP-dependent flavin mono-oxygenase enzyme [12]. All mono-oxygenases include one atom of molecular oxygen in the corresponding substrate, while the other oxygen atom is reduced to H_2_O by a hydrogen donor, which is different for each enzyme. In addition to the hydroxylation of phenol, which is the preferred substrate of the phenol hydroxylase, this enzyme can catalyze the hydroxylation of hydroxyl-, amino-, halogen-, or methyl-substituted phenols [12]. Chlorophenol and (choloromethyl) phenol hydroxylases are employed to convert the substrates into chlorinated catechols or chloromethylcatechols, which play a key role in ortho- or meta-pathways. Phenol hydroxylase also catalyzes the conversion of alkylphenols into alkylcatechols. The aromatic ring of catechol is further opened by catechol 1,2-dioxygenase, leading to the formation of succinyl-CoA and acetyl CoA, or by catechol 2,3-dioxygenase, leading to the formation of pyruvate and acetaldehyde. Moreover, long-chain alkylphenols, nitrophenols, chloronitrophenols, and bisphenols require specific peripheral degradation pathways [91]. The bacterial degradation of nitrophenols can start with mono-oxygenation (a mono-oxygenase catalyzes the nitro groups elimination as a nitrite ion by adding an oxygen atom), the reduction of the nitro group (a nitroreductase catalyzes the nitrogen group reduction into amino groups or hydroxylamine), reductive dehalogenation from halonitrophenol compounds (a reductive dehalogenase removes the halogen atom, following the oxidative removal of the nitro group), and dioxygenation (insertion of two hydroxyl groups with the removal of the nitro group as nitrite ion) [122,123,124].

Some nitrophenol-degrading bacteria have been isolated by utilizing media enriched in mononitrophenols, halonitrophenols, or polynitrophenols. Among the isolated strains were *Arthrobacter nitrophenolicus* sp. [126], *Burkholderia* sp. RKJ [127], *Pseudomonas* sp. [122], *Rhodotorula glutinis* [128], *Sphingomonas* sp. [122], and *Paraburkholderia* [129].

In Mediterranean countries, OWW has been widely studied due to its recalcitrant nature that is related to high polyphenol content. For example, Maza-Marquez et al. [26] demonstrated that the phenolic pollutants found in an OWW storage basin could be treated using specific bacterial taxa, such as *Firmicutes* and *Clostridiales*. Muñoz-Palazon et al. [24] observed that the microbial communities in AGS reactors treating high polyphenol concentrations were dominated by *Lampropedia*, *Acinetobacter*, *Arenimonas*, *Pseudomonas,* and *Corynebacterium*.

*Eukarya* microorganisms are widespread in environmental and engineering niches and play an essential role in the bioremediation field. Many studies have focused on phenolic compound degradation by fungi due to their ability to use organic pollutants as substrates for their growth. Some *Ascomycota* members have been isolated in systems designed for the treatment of OMWW with polyphenols as the sole carbon source, including *Aspergillus niger*, *Penicillium* sp., *Fusarium* sp., and *Alternaria* sp. [59]. The *Basidiomycota* phylum is the major fungal group that has members able to degrade toxic compounds due to their rich production of tyrosinases, laccases, and peroxidases [130]. Tyrosinases oxidate both monophenols and 1,2-diphenols (catechols) to quinones; laccases of a low-molecular-weight use 1,4-diphenols as substrate, which acts as monophenols and polyphenols; peroxidases are oxidoreductases that catalyze peroxide reduction and the concomitant oxidation of various substrates, including phenols [131,132]. These fungal enzymes are widely employed in industrial processes due to their role in improving organoleptic properties. For this reason, some authors highlighted the need for improved knowledge about the employment of wild-type and bioengineered fungi to obtain these enzymes of industrial interest [130,133].

In addition to bacteria and fungi, microalgae have also been recognized for their high capability to resist toxic compounds due to their extraordinary ability to produce extracellular laccases that allow for the biodegrading and transforming of aromatic compounds [134,135]. Microalgae can biotransform, mineralize, and remove compounds under phototrophic or mixotrophic conditions. Some phylotypes, such as *Chlorella* sp., can remove polyphenols under light or dark conditions [136,137]; moreover, it has been reported that *Scenedesmus quadricauda* and *Ankistrodesmus braunii* biotransform polyphenols in simpler compounds [138].

The immense diversity of the metabolic pathways and resistance of prokaryotic and eukaryotic microorganisms allows for the development of various strategies for biotechnological tools in order to provide a high-quality water effluent. However, further combined efforts among chemical, engineering, and microbiological areas have to be made to obtain higher yields via the microorganisms dwelling in engineering systems.

## 7. Conclusions

The biological treatment field has made immeasurable efforts to improve the feasibility and efficiency of the best available techniques for treating contaminated waters with high polyphenol content. From a classical engineering perspective, water treatment has always been more effective using physical-chemical technologies than biological approaches. However, this review provides a comprehensive reading on the success of combining biological, chemical, and engineering aspects to achieve polyphenol removal in water bodies. The main cause of inefficiency in biological water treatment lies in the lack of knowledge about (a) the characteristics of the influent, (b) the selection of the best fit technology, and (c) the optimum operational parameters to promote the biochemical processes. This review emphasizes the important role of microorganisms in these biotechnological processes, discarding the concept of reactors as ‘black boxes’, which exclusively evaluate the mass balance. Biotechnological approaches operated in a precise direction might improve the quality of the treated water in an economically and environmentally sustainable context.

The most decisive parameters for selecting the optimum biological technology for degrading pollutants and amending contaminated water are reported hereafter:

(a) The concentration and nature of the polyphenols (variability of composition);
(b) The presence of other toxic and recalcitrant compounds in the raw water matrix;
(c) The carbon, nitrogen, and phosphorous concentration;
(d) The water quality requirements for the treated water;
(e) Logistics: the operational and economic possibilities for its implementation.

## Figures and Tables

**Table 1:** Phenol levels in industrial wastewaters [1,14,26,27,28].

Type of Industry	Range of Total Phenol Concentration (mg·L^−1^)
Rubber	3–10
Leather	4–6
Ferrous	5–9
Pulp and paper	22
Fiberglass	40–2564
Petroleum-processing plant	40–185
Wood Preserving	50–953
Fabric	100–150
Petrochemical	200–1200
Coke ovens (without dephenolization)	300–3900
Olive washing	400–1120
Agri-food (winery, oil)	400–10,700
Phenolic resins	1270–1345
Coal conversion	1700–7000

## Data Availability

Not applicable.
